# Neuroprotective effects of seaweeds against 6-hydroxidopamine-induced cell death on an *in vitro* human neuroblastoma model

**DOI:** 10.1186/s12906-018-2103-2

**Published:** 2018-02-14

**Authors:** Joana Silva, Celso Alves, Susete Pinteus, Susana Mendes, Rui Pedrosa

**Affiliations:** 1 MARE – Marine and Environmental Sciences Centre, ESTM, Instituto Politécnico de Leiria, 2520-641 Peniche, Portugal; 2 Faculty of Veterinary, University of Santiago de Compostela, 27002 Lugo, Spain

**Keywords:** Parkinson’s disease, Substantia nigra, Mitochondrial membrane potential, Caspase – 3 activity, Oxidative stress, Apoptosis, Marine natural bioactive compounds

## Abstract

**Background:**

Parkinson’s disease (PD) is a progressive neurodegenerative disorder of the central nervous system. Although the causes of PD pathogenesis remain incomplete, some evidences has suggested that oxidative stress is an important mediator in its pathogenesis. The aim of this study was to evaluate the protective effects of seaweeds with high antioxidant activity on 6-hydroxydopamine (6-OHDA)-induced neurotoxicity in the human neuroblastoma cell line SH-SY5Y, as well as the associated intracellular signaling pathways.

**Methods:**

Cell viability studies were assessed by 3-(4,5-dimethylthiazol-2yl)-2,5-diphenyltetrazolium (MTT) bromide assay and the intracellular signaling pathways analyzed were: hydrogen peroxide (H_2_O_2_) production, changes in the mitochondrial membrane potential and Caspase-3 activity.

**Results:**

Exposure of SH-SY5Y cells to 6-OHDA (10–1000 μM) reduced cell’s viability in a concentration and time-dependent manner. The data suggest that the cell death induced by 6-OHDA was mediated by an increase of H_2_O_2_ production, the depolarization of mitochondrial membrane potential and the increase of Caspase-3 activity. Extracts from *S. polyshides, P. pavonica, S. muticum*, *C. tomentosum* and *U. compressa* revealed to efficiently protect cell’s viability in the presence of 6-OHDA (100 μM; 24 h). These effects appear to be associated with the reduction of H_2_O_2_ cell’s production, the protection of mitochondrial membrane’s potential and the reduction of Caspase-3 activity.

**Conclusions:**

These results suggest that seaweeds can be a promising source of new compounds with neuroprotective potential.

## Background

Currently with the increasing of life expectancy and the demographic changes in population, neurodegenerative diseases, such as Alzheimer’s and Parkinson’s disease (PD) are becoming frighteningly common [1, 2]*.*

PD is a progressive neurodegenerative disorder of unknown etiology that is characterized by a progressive loss of dopaminergic neurons in *substantia nigra pars compacta* (SNpc) that underlie characteristic motor symptoms such as rigidity, tremor, slowness of movement, and postural abnormalities [3]. Neuropathology of PD includes insufficient striatal dopamine formation and activity, arising from the death of dopaminergic neurons in SNpc region of the brain. Although the causes of PD pathogenesis remains incomplete, considerable evidences from human and animal studies have suggested that many pathological mechanisms such as oxidative stress, mitochondrial and lysosomal dysfunctions, neuroinflammatory processes, and the formation of pathologic inclusions, contribute to neuronal death [4, 5]. In fact, the nigral dopaminergic neurons are rich in reactive oxygen species (ROS) due to the auto-oxidization of dopamine at normal pH producing toxic dopamine-quinone species, superoxide radicals (O_2_^•−^), hydrogen peroxide (H_2_O_2_), and hydroxyl radicals (·^•^OH). This is supported by increased levels of oxidative products of lipids, proteins, and DNA verified in the *substantia nigra* of PD patients [6]. The pharmacologic treatment of PD can be divided into symptomatic and neuroprotective therapies. The aim of symptomatic strategy is to counteract the deficiency of dopamine in the basal ganglia or to block muscarinic receptors. By other side, the neuroprotective therapy aims to slow, block, or reverse the disease progression. However, such therapies are defined as those that slow the underlying loss of dopaminergic neurons. In fact, at this time, there are no completely proven neuroprotective or disease-modifying therapies [5].

The neurotoxin 6-Hydroxydopamine (6-OHDA) is widely used to mimic experimental models of PD since it can selectively damage dopaminergic neurons in vivo and in vitro. 6-OHDA has a similar structure to dopamine and shows high affinity for the dopamine transporter, therefore it selectively destroys dopaminergic/catecholaminergic neurons [7, 8]. Once inside the neuron, 6-OHDA accumulates and undergoes non-enzymatic auto-oxidation, promoting reactive oxygen species formation. Furthermore, 6-OHDA may provoke the inhibition of mitochondrial complexes I and IV, causing the adenosine triphosphate (ATP) depletion. These evidences suggest the hypothesis that oxidative stress and mitochondrial dysfunction are responsible for the cell death induced by 6-OHDA [9, 10]. In addition, human neuroblastoma SH-SY5Y is a dopaminergic neuronal cell line which has been used as an *in vitro* model for the study of PD and to determine the effect of protective and therapeutic agents. These cells have become a popular research cell model for PD due to the high similarity with dopaminergic neurons [11–14]. The increasing evidences that oxidative stress is critically involved in the pathogenesis of PD suggest that pharmacological targeting of the antioxidant machinery may have therapeutic value [15]. In addition, several experiments revealed that therapies including the intake of synthetic and natural antioxidants have shown a protective effect on the degeneration of dopaminergic neurons [6, 16–19]. Moreover, different studies indicate that the intake of dietary food with high antioxidants content can lower the associated risk of PD [20–24].

The marine environment is known as a rich source of chemical structures with numerous beneficial health effects. It is widely accepted that marine natural products provide unusual and unique chemical structures upon which molecular modeling and chemical synthesis of new drugs can be based with greater efficacy and specificity for the treatment of human diseases [25–27]. Among marine organisms, seaweeds have been target of numerous studies that show their potential as a rich source of structurally diverse biologically active compounds with great pharmaceutical and biomedical potential [28]. Recently, several scientific studies have provided an insight into biological activities and neuroprotective effects of marine algae including antioxidant, anti-neuroinflammatory, cholinesterase inhibitory activity and the inhibition of neuronal death suggesting that marine algae have great potential to be used for neuroprotection as part of pharmaceuticals, nutraceuticals and functional foods [28–30]. In line with this, the main aim of the present study was to investigate the protective effects of seaweeds extracts on 6-hydroxydopamine (6-OHDA)-induced neurotoxicity in the human neuroblastoma cell line SH-SY5Y and the intracellular signaling pathways involved in such effects.

## Methods

### Collection and identification of algae extracts

Seaweeds were collect freshly, between April and July of 2013, in Papôa (39°22′09.5″N 9°22′40.4 W), Quebrado (39°22′04.6″N 9°22′26.1 W) and Gamboa (39°21′54.3″N 9°22′22.7″) beaches, Peniche (Portugal) and immediately transported to laboratory. The seaweeds were then washed with seawater to remove epiphytes, detritus and encrusting material. Algae were identified as *Padina pavonica, Sargassum muticum, Saccorhiza polyschides* (Heterokontophyta division); *Codium tomentosum*, and *Ulva compressa* (Chlorophyta division). Identification was performed by Dr. Susete Pinteus, supported by Marine Algae: Biodiversity, Taxonomy, Environmental Assessment, and Biotechnology guide [31]. Finally algae were kept at − 80 °C (Thermo Scientific, Electron Corporation, Waltham, Massachusetts, USA) until extraction process.

### Preparation of seaweed extracts

Freeze dried seaweeds were sequentially extracted in a 1:40 biomass:solvent ratio with methanol (> 99%, VWR, 20,903.368, Fontenay-sous-Bois, France) and dichloromethane (99%, Fischer Scientifc, D/1852/21/, Loughborough, United Kingdom) at constant stirring for 12 h. Liquid-liquid extraction was also performed for the methanolic fraction, using n-Hexane (99%, Fischer Scientifc, D/1852/21/, Loughborough, United Kingdom). The solvents were evaporated in a rotary evaporator (Laborota 4000, Heidolph, Schwabach, Germany) at 40 °C and the biomass obtained was then solubilized in dimethyl sulfoxide (DMSO) (Sigma Aldrich, 274,380, Saint louis, USA) and stored at − 20 °C until further use.

### Cell culture

The experiments were performed on human neuroblastoma (SH-SY5Y cells), from DMSZ bank - German collection of microorganisms and cell cultures (ACC 209) and maintained with Dulbecco’s Modified Eagle’s Medium (DMEM) (Sigma – Aldrich, D8900, Steinheim am Albuch, Germany) supplemented with 20% (*v*/v) of fetal bovine serum (FBS) (Hyclone, SV30160.0, Northumherlan, UK) and 1% of antibiotic/antimycotic commercial solution (Hyclone, SV30079.01, Utah, USA). For subcultures, SH-SY5Y cells were dissociated with tripsin-EDTA (Hyclone, Thermoscientific, SV30031.01, Waltham Massachusetts, USA), split into a 1:3 ratio and subcultured into Petri dishes with 25 cm^2^ growth area. Medium was replaced every 2 days until the cells reached the total confluence (4–5 days of initial seeding). Cells were maintained in the following controlled conditions: 95% of humidified atmosphere, 5% of CO_2_ and constant temperature of 37 °C.

### Evaluation of neurotoxicity effects induced by 6-OHDA on SH-SY5Y cells viability

The neurotoxicity induced by 6-OHDA (Sigma – Aldrich, H4381 Steinheim am Albuch Germany) on SH-SY5Y cells was evaluated in 96 well plates after the cells reached total confluence. The cells were then incubated with different concentrations of 6-OHDA (10, 30, 100, 300 and 1000 μM) during 24 h. The time-course effects of 6-OHDA (100, 300 and 1000 μM) were also studied after 12, 24 and 48 h of incubation. The solutions were previous prepared in culture medium without FBS and sterile filtered (0.2 μm) (Whatman, Maidstone, UK). The effects were assessed by a colorimetric assay (570 nm) based on the conversion of tetrazolium salts (MTT) (Amresco, 0793-1G, Solon, USA) to blue formazan products by active mitochondria [32, 33]. Results were expressed in percentage of control and as IC_50_ (concentration causing 50% of cell viability reduction) where applicable.

### Cytotoxicity and protective effects of seaweeds extracts on neurotoxicity induced by 6-OHDA

A previous work from our work group screened twenty seven seaweeds for their antioxidant potential, revealing 12 extracts with high antioxidant activity [34]. Within these, only 6 fractions didn’t exhibit toxicity on SH-SY5Y cells (data not shown) being selected for the neuroprotective assays, namely: methanolic extracts – *Sargassum muticum*, *Sacchorhiza polyshides*, *Padina pavonica*; dichloromethane extracts – *Sargassum muticum*, *Codium tomentosum* and *Ulva compressa*. The neuroprotective effect of seaweeds extracts on SH-SY5Y cells in the presence of 6-OHDA was tested after cells reached total confluence in 96 well plates. Cells were incubated with 6-OHDA (100 μM) and seaweeds extracts (1 mg/mL) during 24 h.

#### MTT method

The solutions were previously prepared in culture medium without FBS and sterile filtered (0.2 μm). The effects were estimated by colorimetric assay (570 nm) based on the conversion of tetrazolium salts (MTT) to a blue formazan product by active mitochondria [32, 33]. Results were expressed in percentage of control.

#### Calcein –AM method

This method is based on the fluorophore calcein-AM (Invitrogen, C31100MP, Waltham, Massachusetts, USA). The calcein, in its natural form, exhibits fluorescence; however once esterified (calcein-AM) loses this feature and gain the ability to penetrate cell’s membranes. Within this, the esterase existing in the cytoplasm break the esters links, restoring calcein natural form. For esterases to have a normal activity, cells must be viable, therefore, the greater is the fluorescence intensity greater is the number viable cells [35]. The procedure was adapted from Pedrosa and Soares-da-Silva (2002) [36]. Cells cultured in 96 well plates were incubated with 6-OHDA (100 μM) and seaweeds extracts that exhibited protective activities (1 mg/mL) during 24 h. The solutions were previously prepared in culture medium without FBS and sterile filtered (0.2 μm). Briefly, cells were washed twice with 200 μl of Hank’s buffer and loaded with 100 μl of calcein (2 μM). The 96 well plates were incubated at room temperature protected from light for 30 min and the fluorescence intensity of eight independent experiments were measured by a microplate reader (Biotec, Synergy H1 Hybrid Reader) at wavelengths of 490 nm (excitation) and 520 nm (emission) scanning all of each well surface. The results were expressed in percentage of control of the fluorescence scan read in eight independent wells. After the reader the cells were washed again with 200 μl Hank’s buffer and finally was added 100 μl of Hank’s buffer to each situation. The effects were observed in a fluorescence inverted microscope (ZEISS Axio, VERT. A1, equipped with a AxioCam MRC-ZEISS camera, München, Germany). The images presented are representative of one well center-point of each situation tested.

### Quantification of hydrogen peroxide (H_2_O_2_) production

Quantification of H_2_O_2_ was performed using the “Amplex ™ Red hydrogen peroxide Assay” Kit (Life Tecnologies, A22188, Camarillo, USA). The amplex red is a fluorophore that evidence a low basal fluorescence which reacts in with H_2_O_2_ in a 1:1 ratio. This reaction is initiated in the presence of horseradish peroxidase and successive reactions occur leading to the appearance of highly fluorescent product, designated resofurin [37]. H_2_O_2_ production was quantified in SH-SY5Y cells after 12 h of treatment with 6-OHDA (100 μM) in the absence or presence of seaweeds extracts (1 mg/mL). The variation of H_2_O_2_ production was accompanied in real-time along 60 min at room temperature. The fluorescence intensity was measured at wavelengths of 590 nm (excitation) and 530 nm (emission). The levels of H_2_O_2_ were calculated by the slope of the linear phase of fluorescence curve and the results were expressed in percentage of control.

### Mitochondrial membrane potential (MMP)

MMP was determined using the fluorescent probe, JC-1 (Molecular Probes, T3168, Eugene, Oregon, USA). SH-SY5Y cells were treated with 6-OHDA (100 μM), 3 and 6 h, in the absence or presence of seaweeds extracts (1 mg/mL). After this period, the culture medium was removed and the cells were washed with Hank’s buffer and incubated 15 min at 37 °C with JC-1 (3 μM). JC-1 probe was then removed and cells washed with Hank’s buffer. The formation of JC-1 aggregates (490 nm of excitation and 590 nm of emission) and the monomeric form of JC-1 (490 nm of excitation and 530 nm of emission) was accompanied simultaneously in the plate reader during 30 min. Results were expressed as the ratio of the monomers/aggregates of JC-1 in percentage of control.

### Caspase-3 activity

Caspase-3 activity was assessed using “Caspase Assay kit” (Sigma, Casp3f, Saint Louis, USA). Cells were cultured in 6-well plates and treated with 6-OHDA (100 μM) 6 h in the presence or absence of seaweeds extracts (1 mg/mL). After this period the culture medium was removed, the cells were washed twice with Hank’s buffer and collected by centrifugation (8000 rpm, 10 min, 4 °C). The pellets were resuspended in 100 μL of lysis buffer and incubated 20 min on ice. In order to separate the content of intracellular cytoplasmic organelles and cells membranes, centrifugation took place at 14000 rpm during 20 min at 4 °C. Thereafter, 5 μL of the obtained supernatant was place in to a 96-well plate where it was added 200 μL of a substrate solution which was prepared following the manufacturer’s instructions. In this assay, the Caspase-3 activity is accessed by measuring the fluorescence released by the fluorophore rhodamine 110. When attached to the amino acid sequence, this fluorophore have a basal fluorescence, however, when detached from the amino acid sequence, rhodamine 110 is highly fluorescent. Caspase-3 is specific for cleaving the rhodamine-aminoacid solution (substrate) resulting in the fluorescent form of rhodamine 110, the product. This reaction was followed at wavelengths of 496 nm (excitation) and 520 nm (emission) along 60 min at room temperature. Caspase-3 activity was calculated by the slope of the linear phase of the fluorescence resulting from the rhodamine 110 accumulation and expressed in arbitrary fluorescence units per mg protein per minute (Δfluorescence (u.a)/mg of protein/min).

### Statistical analysis

One-way analysis of variance (ANOVA) with Dunnett’s multiple comparison of group means were employed to determine significant differences relatively to the control treatment [38]. All other post-hoc analyses were accessed through Tukey test. All data were checked for normality and homoscedasticity. Comparisons concerning variables, which did not meet variance or distributional assumptions, were carried out with Kruskal-Wallis non-parametric tests [38]. Where applicable, results are presented as mean ± standard error of the mean (SEM). Differences were considered statistically significant at level of 0.05 (that is, *p* < 0.05). All calculations were performed using IBM SPSS Statistics 21 (IBM Corporation, Armonk, NY, USA) and GraphPad v5.1 (Graphpad Software, Inc. La Jolla, CA, USA). The determination of IC_50_ was calculated by the analysis of non-linear regression using GraphPad Prism software with the eq. Y = 100/ (1 + 10 ^(X - LogIC50)^) equation.

## Results

### Neurotoxic effects induced by 6-OHDA on SH-SY5Y cells

**Fig. 1:**
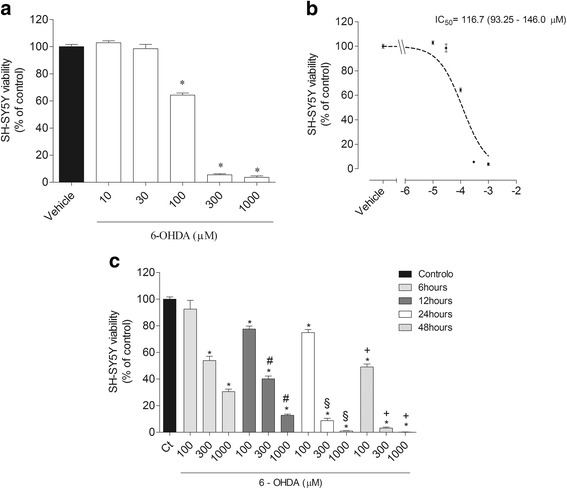
Concentration dependent effect of 6-OHDA (10–1000 μM) on SH-SY5Y cells viability (% of control) after 24 h of incubation (**a**); IC_50_ obtained from different concentrations of 6-OHDA (100-1000 μM) (**b**); Effect of 6 - OHDA (100–1000 μM) on SH-SY5Y cells viability (% of control) after different times of incubation 6, 12, 24 and 48 h (**c**). Results were obtained by the MTT method. Values are mean ± SEM (*n* = 16). Symbols represent statistically significant differences (*p* < 0.05, ANOVA, Tukey test) when compared to: *vehicle; ^#^6 h; ^§^12 h; ^+^ 24 h

SH-SY5Y cells were exposed to different concentrations of 6-OHDA (10–1000 μM) during 24 h. As can be observed in the Fig. 1, 6-OHDA induced a concentration-dependent effect on the viability of SH-SY5Y cells with an IC_50_ of 116.7 μM (93.25–146.6). The highest neurotoxicity effect was obtained at 300 μM and 1000 μM with a cell viability reduction of more than 80%. For the concentrations (100–1000 μM) that exhibited neurotoxicity on SH-SY5Y cells was defined the time-course effects after 6, 12, 24 and 48 h. All the tested concentrations of 6-OHDA showed a time-dependent effect (Fig. 1b).

### Neuroprotective effect of seaweeds on SH-SY5Y cells exposed to 6-OHDA

**Fig. 2:**
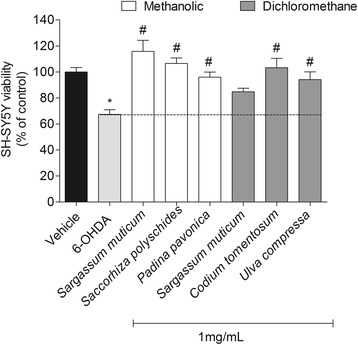
Neuroprotective effects of seaweeds extracts (1 mg/mL) on SH-SY5Y cells exposed to 6-OHDA (100 μM). Determination of cell viability by the MTT method (% control) after 24 h of incubation. Values are mean ± SEM (n = 16). Symbols represent statistically significant differences (*p* < 0.05, ANOVA, Dunett’s test) when compared to: ^*^vehicle. ^#^ to 6-OHDA

**Fig. 3:**
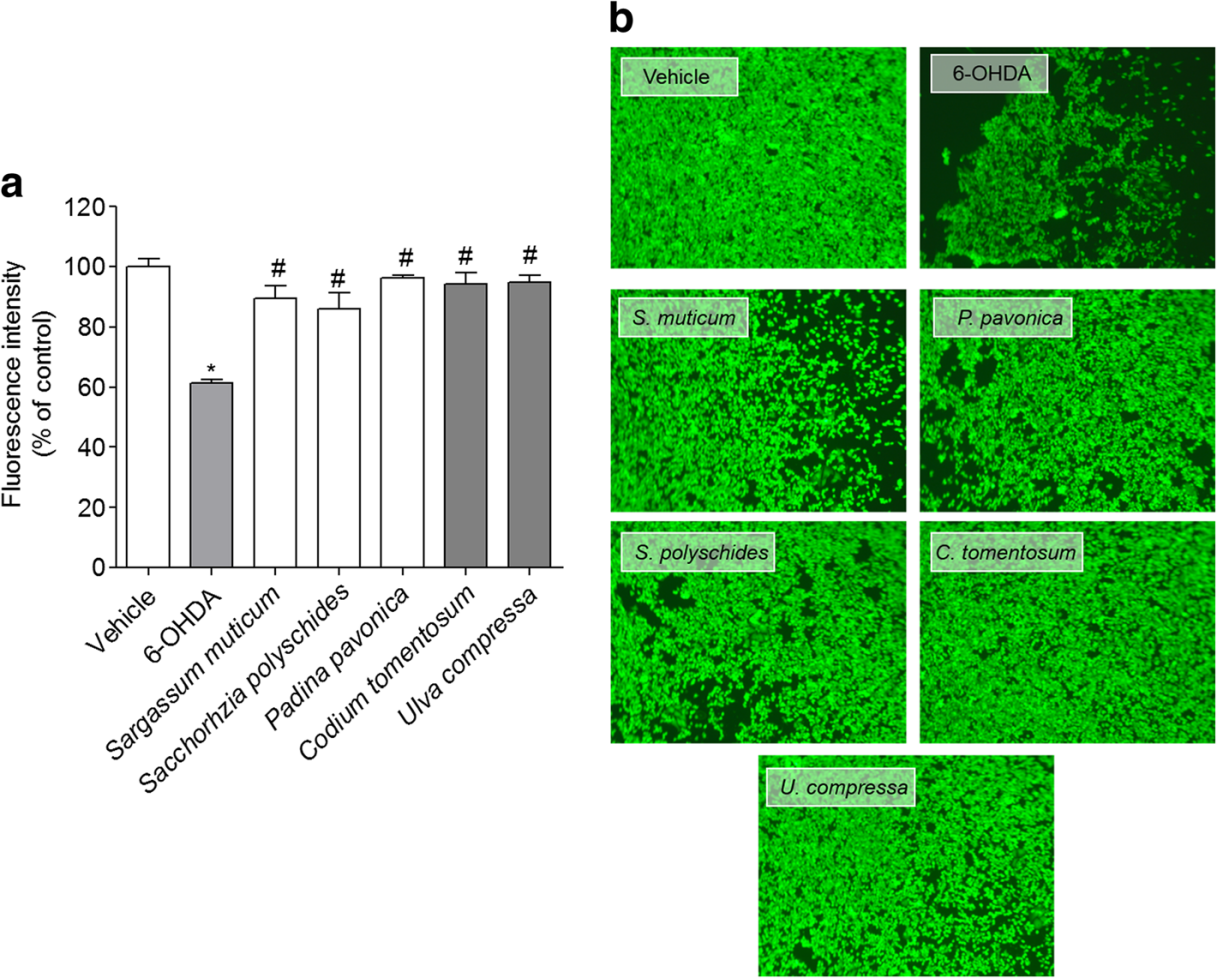
Neuroprotective effects of seaweeds extracts (1 mg/mL) on SH-SY5Y cells exposed to 6-OHDA (100 μM) revealed by Calcein-AM method **a**) Read of fluorescence (% control) after 24 h of incubation. Values are mean ± SEM (*n* = 8). Symbols represent statistically significant differences (*p* < 0.05, ANOVA, Dunett’s test) when compared to: ^*^ vehicle. ^#^ to 6-OHDA. **b** Observation by fluorescence microscopy of the effects induced by 6-OHDA and seaweeds extracts. The images are representative of each treatment done and correspond to the center-point of one well. Methanolic extract: *Sargassum muticum*, *Padina pavonica* and *Saccorhiza polyschides*; Dichloromethane extract: *Codium tomentosum* and *Ulva compressa*

The exposition of SH-SY5Y cells to 6-OHDA (100 μM) led to a reduction of about 35% (67.40 ± 3.56 of viable cells) of cell’s viability. However, when 6-OHDA was incubated with seaweeds (1 mg/mL), all extracts, with exception for the dichloromethane extract of *S. muticum* exhibited capacity to totally blunt the toxicity induced by 6-OHDA after 24 h of incubation (Fig. 2). On the other hand, the dichloromethane extract of *S. muticum* (84.73 ± 2.73% of viable cells) did not showed statistically significant differences when compared to 6-OHDA situation (ANOVA, *p* > 0.05). These results were confirmed both by the Calcein -AM assay that showed that all extracts protected cells from the toxic effects induced by 6-OHDA (Fig. 3a) and by the representative fluorescent images of 6-OHDA treatment in the presence or in the absence of the seaweeds extracts (Fig. 3b).

### Cellular mechanisms associated to the neurotoxicity induced by 6-OHDA on SH-SY5Y cells in the presence or absence of seaweeds extracts

#### Production of H_2_O_2_

In order to understand if the neurotoxicity induced by 6-OHDA and the neuroprotection evidenced by seaweeds extracts on cell viability of SH-SY5Y was associated with oxidative stress, the H_2_O_2_ production was quantified.

**Fig. 4:**
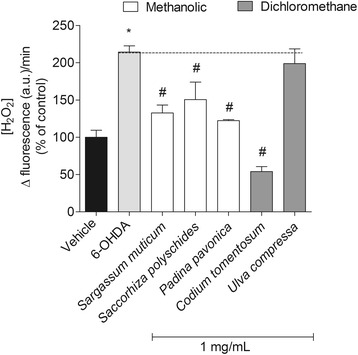
Levels of hydrogen peroxide (H_2_O_2_) produced by SH-SY5Y cells after 12 h of incubation with 6-OHDA (100 μM) in presence or absence of seaweeds extracts (1 mg/mL). H_2_O_2_ was quantified fluorimetrically using the “™ Amplex red hydrogen peroxide assay” kit. Symbols represent statistically significant differences (*p* < 0.05, ANOVA, Dunett’s test) when compared to: ^*^vehicle. ^#^ to 6-OHDA

The exposition of SH-SY5Y cells to 6-OHDA (100 μM) led to an increase of more than twice the levels of H_2_O_2_ comparing with vehicle (Fig. 4). Moreover, when the cells were incubated with 6-OHDA in the presence of seaweeds extracts (1 mg/mL) the levels of H_2_O_2_ decreased partially or totally when compared with 6-OHDA. The highest decrease was carried out by the dichloromethane extract of *Codium tomentosum* (54.07 ± 6.66% of control) when compared with 6-OHDA (214.26 ± 8.46% of control) and vehicle (100.00 ± 9.48% of control). Within the extracts with neuroprotective potential, *Ulva compressa* was the only extract that did not exhibited capacity to decrease the H_2_O_2_ production induced by 6-OHDA.

### Mitochondrial membrane potential (MMP)

**Fig. 5:**
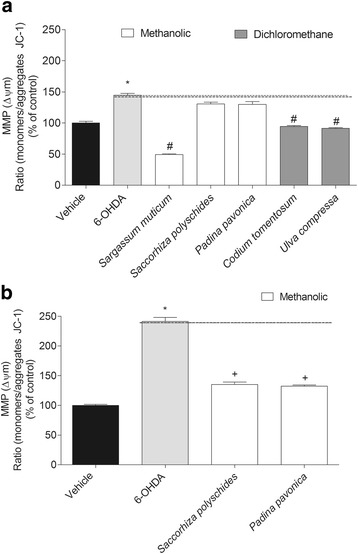
6-OHDA (100 μM) effects in the presence or absence of seaweeds extracts (1 mg/mL) in mitochondrial membrane potential of SH-SY5Y cells after 3 h (**a**) and 6 h (**b**) of incubation. The results were obtained by the ratio between the monomers/aggregates of JC-1. The values in each column represent the mean ± standard error of the mean (SEM) of 3 or 4 independent experiments. Symbols represent statistically significant differences (*p* < 0.05, ANOVA, Dunett’s test) when compared to: ^*^vehicle. ^#^ to 6-OHDA

The incubation of SH-SY5Y cells with 6-OHDA (100 μM – 3 h) induced a strong depolarization of the MMP when compared with vehicle (Fig. 5a). Moreover, this effect was also time-dependent, since after 6 h of incubation with 6-OHDA the depolarization increased twice when compared with the 3 h exposure. During the treatment with seaweeds (3 h) it was possible to observe a noticeable preventive effect of *S. muticum*, *C. tomentosum* and *U. compressa* extracts in the depolarization induced by 6-OHDA. On the other hand, the extracts of *S. polyschides* and *P. pavonica* only prevented the effects induced by 6-OHDA after 6 h of incubation (Fig. 5b).

#### Caspase-3 activity

**Fig. 6:**
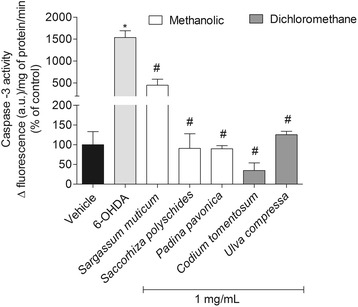
6-OHDA (100 μM) effects in the presence or absence of seaweeds extracts (1 mg/mL) on Caspase-3 activity of SH-SY5Y cells after 6 h of treatment. The activity was quantified by the slope of the linear phase accumulation of Rhodamine 110 (between 20 and 40 min). The results are presented in arbitrary units of fluorescence per mg protein. The values in each column represent the mean ± standard error of the mean (SEM) from 3 to 4 experiments. Symbols represent statistically significant differences (*p* < 0.05, ANOVA, Dunett’s test) when compared to: ^*^vehicle. ^#^ to 6-OHDA

In order to understand if the cell death promoted by 6-OHDA is mediated by apoptosis, it was decided to study the Caspase-3 activity, since it is an important biomarker in this process. The results showed a noticeable increase of Caspase-3 activity when SH-SY5Y cells were treated with 100 μM of 6-OHDA (1536.72 ± 154.76% of control) comparing with vehicle (100 ± 33.24% of control). Furthermore, when cells were incubated with 6-OHDA and seaweeds extracts (1 mg/mL) it was possible to detect a reduction of Caspase-3 activity when compared with 6-OHDA. The extracts from *C. tomentosum*, *S. polyschides*, *P. pavonica* and *U. compressa* completely inhibited the stimulation of Caspase-3 by 6-OHDA. *S. muticum* also revealed this capacity but the effects were not so marked (Fig. 6).

## Discussion

PD is a neurodegenerative disease of the central nervous system characterized by a progressive loss of dopaminergic neurons that underlie the characteristic motor symptoms. This disease still doesn’t have an affective cure and therefore exists an increasing interest in the development of more selective and effective therapeutic agents in order to prevent or slow down the neurodegeneration progression [15, 18, 25, 26, 39]. Although the causes PD pathogenesis remains incomplete, considerable evidences from human and animal studies have suggested that mechanisms such as oxidative stress, mitochondrial and lysosomal dysfunctions, neuroinflammatory processes, and the formation of pathologic inclusions contributes to neuronal death in PD [4, 5]. In line with this, the present study was designed to evaluate the protective effects of seaweeds on SH-SY5Y cells exposed to the toxicity of 6-OHDA.

The neurotoxicity induced by 6-OHDA on SH-SY5Y cells has been previously reported by different authors, consequently, this model has been widely used to mimic experimental models of PD [5, 6, 11, 40]. According to previous studies, once inside the neurons, 6-OHDA accumulates and undergoes a non-enzymatic auto-oxidation, promoting the reactive oxygen species formation (e.g. superoxide radical, hydrogen peroxide, quinones and hydroxyl radicals) and inhibit the mitochondrial complexes I and IV, causing adenosine triphosphate (ATP) depletion. All these supports the hypothesis that oxidative stress and mitochondrial dysfunction may be responsible for the cell death [5, 41, 42]. Moreover, these two events are described as being entirely related, since the occurrence of mitochondrial dysfunction can lead to ATP depletion, inducing irreversible effects on the cellular processes, leading to the formation of free radicals. Consequently, the loss of mitochondrial transmembrane potential can result in the rupture of the outer mitochondrial membrane and in the release of pro-apoptotic proteins from the nucleus leading to cell death through activation of the intrinsic apoptosis pathway [43–45]. Our results are entirely according with these facts, since the observed reduction on SH-SY5Y cells viability was accompanied by an increase of H_2_O_2_ production, depolarization of mitochondrial membrane potential and an increase of Caspase-3 activity, suggesting that cell death induced by the 6-OHDA treatment was mediated by these mechanisms. Moreover, our results are supported by Esmaeili-Mahani and co-workers [11] that observed a significant increase of intracellular ROS, activated Caspase 3, Bax/Bcl-2 ratio, cytochrome c as well as DNA fragmentation in 6-OHDA-treated cells. For other side, when SH-SY5Y cells were treatment with 6-OHDA in the presence of seaweeds extracts was possible to see a marked increase of cell’s viability. The data obtained suggests that the protective effects induced by seaweeds extracts result in a reduction of oxidative stress condition (H_2_O_2_ production pathway) and an anti-apoptotic effect (mitochondrial protection and decrease of Caspase-3 activity). Several experiments revealed that therapies including the intake of antioxidants display a protective effect on the degeneration of dopaminergic neurons suggesting that pharmacological targeting of the antioxidant machinery may have therapeutic value [6, 16–19, 46]. According with this, the neuroprotective effects of seaweeds can be mediated by the antioxidants molecules present in their extracts, since these were selected by having a high antioxidant capacity. Among marine organisms, seaweeds are an interesting source of new compounds with antioxidant activity and neuroprotective potential. They are subjected to periods of immersion and emersion being exposed to rapid variations of light, UV rays and different oxygen (O_2_) and carbon dioxide (CO_2_) concentrations, factors that are related with oxidizing effects. This situation stimulates the production of antioxidant defenses, such as the production of phenolic compounds [47, 48]. The protective effects exhibited by seaweeds belonging to the brown algae group (*S. muticum*, *S. polyschides* and *P. pavonica*) can be associated with the presence of phlorotannins (molecules produced exclusively by brown algae). Phlorotannins, are phenolic compounds with a strong antioxidant capability [49, 50] suggesting that these may be responsible by the observed reduction of H_*2*_O_2_ levels. In fact, seaweeds have revealed to produce a high diversity of compounds with antioxidant activity. *Codium tomentosum*, a green seaweed also revealed an oxidative-stress protective effect. Celikler and co-workers [51] demonstrated that *C. tomentosum* extracts have strong anti-oxidative activity which can explain the highest reduction of the H_2_O_2_ production observed in presence of this extract.

On the other hand, several studies reported that the neuroprotection effect of different compounds is normally mediated by the prevention of mitochondrial depolarization, the reduction of ROS levels and the inhibition of apoptotic process leading to an increase of cell’s viability [11, 52, 53]. In our study was also possible to observe these effects, since the increase of cell’s viability by seaweeds extracts seems to be mediated by the reduction of H_2_O_2_ levels, the protection of mitochondrial membrane potential and the inhibition of Caspase-3 activity. According to our results, several other compounds of natural origin such as, astaxanthin (obtained from *Haematococcus pluvialis*) and 11-dehydrosinulariolide (obtained from the coral *Sinularia flexibilis*) have exhibited anti-apoptotic effects, notably by decreasing the Caspase-3 expression and cytochrome c in SH-SY5Y cells when treated 6-OHDA. Similarly, the neuropeptide orexin-A, exhibit anti-apoptotic effects by the same mechanisms [6, 11, 31]. In line with our findings, Jhamandas and co-workers [54] showed neuroprotective activity of a fucoidan sulfated polysaccharide, isolated from the brown seaweed *Fucus vesiculosus*, through the ability to protect neuronal death in rats treated with Aβ1–42 the by inhibition of Caspase-3 in an Alzheimer’s disease model. Although not directly related to the disease addressed in this work, the effects induced by fucoidan demonstrate the potential of seaweeds as source of new neuroprotective molecules.

## Conclusions

In conclusion, the seaweeds extracts with high antioxidant capacity analyzed in this study showed to be a promising source of new compounds with neuroprotective potential revealing capacity to increase the SH-SY5Y cell’s viability by the reduction of H_2_O_2_ levels, the protection of mitochondrial membrane potential and the inhibition of Caspase-3 activity, reducing the neurotoxic effects induced by 6-OHDA.

## Abbreviations

•OH: Hydroxyl radical
6-OHDA: 6- Hydroxidopamine
ANOVA: Analysis of variance
ATP: Adenosine trisphosphate
*C. tomentosum*: *Codium tomentosum*
CO_2_: Carbon dioxide
DMEM: Dulbecco’s modified Eagle medium
FBS: Fetal bovine serum
H_2_O_2_: Hydrogen peroxide
JC-1: 5,5′,6,6′-tetrachloro-1,1′,3,3′-tetraethylbenzimi- dazolylcarbocyanine iodide
MTT: 3-(4,5-dimethylthiazol-2yl)-2,5-diphenltetrazolium bromide
O_2_: Oxigen
O2•^−^: Superoxide radical
*P. pavonica*: *Padina pavonica*
PD: Parkinson Disease
ROS: Reactive oxygen species
*S. muticum*: *Sargassum muticum*
*S. pPolyschides*: *Saccorhiza polyschides*
SH-SY5Y: Human neuroblastoma cell line
SNpc: Substancia nigra par compact
*U. compressa*: *Ulva compressa*

## References

[1] Skovronsky DM, Lee VM-Y, Trojanowski JQ (2006). Neurodegenerative diseases: new concepts of pathogenesis and their therapeutic implications. Annu Rev Pathol Mech Dis.

[2] García-Ayllón M-S, Cauli O, Silveyra M-X, Rodrigo R, Candela A, Compañ A, Jover R, Pérez-Mateo M, Martínez S, Felipo V (2008). Brain cholinergic impairment in liver failure. Brain.

[3] Bernheimer H, Birkmayer W, Hornykiewicz O, Jellinger K, Seitelberger F (1973). Brain dopamine and the syndromes of Parkinson and Huntington clinical, morphological and neurochemical correlations. J Neurol Sci.

[4] Tolleson CM, Fang JY (2013). Advances in the mechanisms of Parkinson’s disease. Discov Medicine.

[5] Cunha MP, Martín-de-Saavedra MD, Romero A, Parada E, Egea J, del Barrio L, Rodrigues ALS, López MG (2013). Protective effect of creatine against 6-hydroxydopamine-induced cell death in human neuroblastoma SH-SY5Y cells: involvement of intracellular signaling pathways. Neuroscience.

[6] Ikeda Y, Tsuji S, Satoh A, Ishikura M, Shirasawa T, Shimizu T (2008). Protective effects of astaxanthin on 6-hydroxydopamine-induced apoptosis in human neuroblastoma SH-SY5Y cells. J Neurochem.

[7] Duty S, Jenner P (2011). Animal models of Parkinson’s disease: a source of novel treatments and clues to the cause of the disease. Br J Pharmacol.

[8] Tieu K (2011). A guide to neurotoxic animal models of Parkinson’s disease. Cold Spring Harb. Perspect. Med..

[9] Perier C, Vila M (2012). Mitochondrial biology and Parkinson's disease. Cold Spring Harb Perspect Med.

[10] Dias V, Junn E, Mouradian MM (2013). The role of oxidative stress in Parkinson's disease. J Park Dis.

[11] Esmaeili-Mahani S, Vazifekhah S, Pasban-Aliabadi H, Abbasnejad M, Sheibani V (2013). Protective effect of orexin-a on 6-hydroxydopamine-induced neurotoxicity in SH-SY5Y human dopaminergic neuroblastoma cells. Neurochem Int.

[12] Segal RA, Greenberg ME (1996). Intracellular signaling pathways activated by neuropathic factors. Annu Rev Neurosci.

[13] Lotharius J, Dugan LL, O’malley KL (1999). Distinct mechanisms underlie neurotoxin-mediated cell death in cultured dopaminergic neurons. J Neurosci.

[14] Xie HR, Hu LS, Li GY (2010). SH-SY5Y human neuroblastoma cell line: in vitro cell model of dopaminergic neurons in Parkinson’s disease. Chin Med J.

[15] Lou H, Jing X, Wei X, Shi H, Ren D, Zhang X (2014). Naringenin protects against 6-OHDA-induced neurotoxicity via activation of the Nrf2/ARE signaling pathway. Neuropharmacology.

[16] Tian L-L, Wang X-J, Sun Y-N, Li C-R, Xing Y-L, Zhao H-B, Duan M, Zhou Z, Wang S-Q (2008). Salvianolic acid B, an antioxidant from salvia miltiorrhiza, prevents 6-hydroxydopamine induced apoptosis in SH-SY5Y cells. Int J Biochem Cell Biol.

[17] Levites Y, Youdim MBH, Maor G, Mandel S (2002). Attenuation of 6-hydroxydopamine (6-OHDA)-induced nuclear factor-kappaB (NF-κB) activation and cell death by tea extracts in neuronal cultures. Biochem Pharmacol.

[18] Mansouri MT, Farbood Y, Sameri MJ, Sarkaki A, Naghizadeh B, Rafeirad M (2013). Neuroprotective effects of oral gallic acid against oxidative stress induced by 6-hydroxydopamine in rats. Food Chem.

[19] Mandel S, Youdim MBH (2004). Catechin polyphenols: neurodegeneration and neuroprotection in neurodegenerative diseases. Free Radic Biol Med.

[20] de Rijk MC, Breteler MB, den Breeijen JH (1997). Dietary antioxidants and parkinson disease: the rotterdam study. Arch Neurol.

[21] Etminan M, Gill SS, Samii A (2005). Intake of vitamin E, vitamin C, and carotenoids and the risk of Parkinson's disease: a meta-analysis. Lancet Neurol.

[22] Gao X, Chen H, Fung TT, Logroscino G, Schwarzschild MA, Hu FB, Ascherio A (2007). Prospective study of dietary pattern and risk of Parkinson disease. Am J Clin Nutr.

[23] Okubo H, Miyake Y, Sasaki S, Murakami K, Tanaka K, Fukushima W, Kiyohara C, Tsuboi Y, Yamada T, Oeda T (2012). Dietary patterns and risk of Parkinson’s disease: a case–control study in Japan. Eur J Neurol.

[24] Miyake Y, Fukushima W, Tanaka K, Sasaki S, Kiyohara C, Tsuboi Y, Yamada T, Oeda T, Miki T, Kawamura N (2011). Dietary intake of antioxidant vitamins and risk of Parkinson’s disease: a case–control study in Japan. Eur J Neurol.

[25] Haefner B (2003). Drugs from the deep: marine natural products as drug candidates. Drug Discov Today.

[26] Murray PM, Moane S, Collins C, Beletskaya T, Thomas OP, Duarte AWF, Nobre FS, Owoyemi IO, Pagnocca FC, Sette LD (2013). Sustainable production of biologically active molecules of marine based origin. New Biotechnol.

[27] Horta A, Pinteus S, Alves C, Fino N, Silva J, Fernandez S, Rodrigues A, Pedrosa R (2014). Antioxidant and antimicrobial potential of the *Bifurcaria bifurcata* epiphytic bacteria. Mar. Drugs.

[28] Pangestuti R, Kim S-K (2011). Neuroprotective effects of marine algae. Mar Drugs.

[29] Ahn B, Moon H, Kim H, Jung H, Choi J (2012). Neuroprotective effect of edible brown alga *Eisenia bicyclis* on amyloid beta peptide-induced toxicity in PC12 cells. Arch Pharm Res.

[30] Jin D-Q, Lim CS, Sung J-Y, Choi HG, Ha I, Han J-S (2006). Ulva Conglobata, a marine algae, has neuroprotective and anti-inflammatory effects in murine hippocampal and microglial cells. Neurosci Lett.

[31] Chen W-F, Chakraborty C, Sung C-S, Feng C-W, Jean Y-H, Lin Y-Y, Hung H-C, Huang T-Y, Huang S-Y, Su T-M (2012). Neuroprotection by marine-derived compound, 11-dehydrosinulariolide, in an in vitro Parkinson’s model: a promising candidate for the treatment of Parkinson’s disease. Naunyn Schmiedeberg's Arch Pharmacol.

[32] Castel J, Lechn G. In vitro methods in pharmaceutical research. In: Academic Press (1st Eds). London; 1996. p. 467.

[33] Rotter BA, Thompson BK, Clarkin S, Owen TC (1993). Rapid colorimetric bioassay for screening of Fusarium mycotoxins. Nat Toxins.

[34] Pinteus S, Silva J, Alves C, Horta A, Fino N, Inês Rodrigues A, Mendes S, Pedrosa R. Cytoprotective effect of seaweeds with high antioxidant activity from the Peniche coast (Portugal). Food Chem. 2016. 10.1016/j.foodchem.2016.09.067 27719954

[35] Hayes W, Kruger C. Hayes principles and methods of toxicology. In: Taylor & Francis Group (6th Eds). Boca Raton; 2014. p. 824.

[36] Pedrosa R, Soares-da-Silva P (2002). Oxidative and non-oxidative mechanisms of neuronal cell death and apoptosis by L-3,4-dihydroxyphenylalanine (L-DOPA) and dopamine. Br J Pharmacol.

[37] Mohanty J, Jaffe JS, Schulman ES, Raible DG (1997). A highly sensitive fluorescent micro-assay of H_2_O_2_ release from activated human leukocytes using a dihydroxyphenoxazine derivative. J Immunol Methods.

[38] Zar JH (2010). Biostatistical analysis.

[39] Kopalli SR, Noh S-J, Koppula S, Suh Y-H (2013). Methylparaben protects 6-hydroxydopamine-induced neurotoxicity in SH-SY5Y cells and improved behavioral impairments in mouse model of Parkinson’s disease. Neurotoxicology.

[40] Tiong CX, Lu M, Bian J-S (2010). Protective effect of hydrogen sulphide against 6-OHDA-induced cell injury in SH-SY5Y cells involves PKC/PI3K/Akt pathway. Br J Pharmacol.

[41] Bové J, Perier C (2012). Neurotoxin-based models of Parkinson’s disease. Neuroscience.

[42] Glinka Y, Tipton K, Youdim M (1996). Nature of inhibition of mitochondrial respiratory complex I by 6-Hydroxydopamine. J Neurochem.

[43] Joza N, Susin SA, Daugas E, Stanford WL, Cho SK, Li CY, Sasaki T, Elia AJ, Cheng H-YM, Ravagnan L (2001). Essential role of the mitochondrial apoptosis-inducing factor in programmed cell death. Nature.

[44] Beal MF (2000). Energetics in the pathogenesis of neurodegenerative diseases. Trends Neurosci.

[45] Onyango IG (2008). Mitochondrial dysfunction and oxidative stress in Parkinson’s disease. Neurochem Res.

[46] Kita T, Asanuma M, Miyazaki I, Takeshima M (2014). Protective effects of Phytochemical antioxidants against neurotoxin-induced degeneration of Dopaminergic neurons. J Pharmacol Sci.

[47] Zandi K, Tajbakhsh S, Nabipour I, Rastian Z, Yousefi F, Sharafian S, Sartavi K (2013). In vitro antitumor activity of *Gracilaria corticata* (a red alga) against Jurkat and molt-4 human cancer cell lines. Afr J Biotechnol.

[48] Ghasemzadeh A, Jaafar HZE, Rahmat A, Wahab PEM, Halim MRA (2010). Effect of different light intensities on Total Phenolics and Flavonoids synthesis and anti-oxidant activities in young ginger varieties (Zingiber Officinale roscoe). Int J Mol Sci.

[49] Kang K, Park Y, Hwang HJ, Kim SH, Lee JG, Shin H-C (2003). Antioxidative properties of brown algae polyphenolics and their perspectives as chemopreventive agents against vascular risk factors. Arch Pharm Res.

[50] Dalle-Donne I, Rossi R, Colombo R, Giustarini D, Milzani A (2006). Biomarkers of oxidative damage in human disease. Clin Chem.

[51] Celikler S, Vatan O, Yildiz G, Bilaloglu R (2009). Evaluation of anti-oxidative, genotoxic and antigenotoxic potency of *Codium tomentosum* Stackhouse ethanolic extract in human lymphocytes in vitro. Food Chem Toxicol.

[52] Kim DW, Ahan SH, Kim TY (2007). Enhancement of arsenic trioxide (As2O3)-mediated apoptosis using berberine in human neuroblastoma SH-SY5Y cells. J Korean Neurosurg Soc.

[53] Luo T, Zhang H, Zhang W-W, Huang J-T, Song E-L, Chen S-G, He F, Xu J, Wang H-Q (2011). Neuroprotective effect of Jatrorrhizine on hydrogen peroxide-induced cell injury and its potential mechanisms in PC12 cells. Neurosci Lett.

[54] Jhamandas JH, Wie MB, Harris K, MacTavish D, Kar S (2005). Fucoidan inhibits cellular and neurotoxic effects of β-amyloid (Aβ) in rat cholinergic basal forebrain neurons. Eur J Neurosci.

